# Hydroxypropyl Cellulose Derived from Sugarcane Bagasse as a Tablet Binder and Drug Delivery Matrix: A Structured Narrative Review of Synthesis, Pharmaceutical Performance, and Sustainability Indicators Relative to Commercial Grades

**DOI:** 10.3390/polym18161963

**Published:** 2026-08-11

**Authors:** Yusdan Yulidan Aulia Nisa, Ida Musfiroh, Amirah Mohd Gazzali, Okta Nama Putra, Taufik Muhammad Fakih, Derina Paramitasari, Karjawan Pudjianto, Muchtaridi Muchtaridi

**Affiliations:** 1 Department of Pharmaceutical Analysis and Medicinal Chemistry, Faculty of Pharmacy, Universitas Padjadjaran, Sumedang 45363, Indonesia; yusdan24001@mail.unpad.ac.id (Y.Y.A.N.); ida.musfiroh@unpad.ac.id (I.M.); taufikmuhammadf@unisba.ac.id (T.M.F.); 2 Metro City Health Department, Metro City 34124, Indonesia; 3 School of Pharmaceutical Sciences, Universiti Sains Malaysia, Gelugor 11800, Penang, Malaysia; amirahmg@usm.my; 4 Doctoral Program of Pharmacy, Faculty of Pharmacy, Universitas Padjadjaran, Sumedang 45363, Indonesia; okta22001@mail.unpad.ac.id; 5 National Research and Innovation Agency (BRIN), Tangerang 15314, Indonesia; 6 Department of Pharmacy, Faculty of Mathematics and Natural Sciences, Universitas Islam Bandung, Bandung 40116, Indonesia; 7 Research Center for Process Technology, National Research and Innovation Agency (BRIN), Bacharuddin Jusuf Habibie Science and Technology Park, South Tangerang 15314, Indonesia; deri003@brin.go.id (D.P.); karj001@brin.go.id (K.P.); 8 Research Collaboration Centre for Radiopharmaceuticals Theranostic, National Research and Innovation Agency (BRIN), Sumedang 45363, Indonesia

**Keywords:** sugarcane bagasse, hydroxypropyl cellulose, pharmaceutical excipient, tablet binder, drug delivery matrix, sustainable excipient, circular bioeconomy

## Abstract

Hydroxypropyl cellulose (HPC) is extensively utilized as a binder and in controlled-release matrices, yet its production predominantly relies on high-purity α-cellulose derived from wood or cotton, which subjects supply chains to sustainability issues and fluctuations in feedstock prices. Annually, sugarcane bagasse, estimated at approximately 490–600 million tons annualy, presents a scalable, residue-based cellulose source for HPC production within circular bioeconomy frameworks. This review compiles findings from 106 peer-reviewed studies on cellulose and HPC derived from bagasse, addressing synthesis methods, structure–property relationships, and pharmaceutical applications. Published studies indicate that HPC derived from bagasse can achieve a degree of substitution (DS 1.87) compared to commonly reported commercial wood-pulp HPC grades (DS 1.8–2.5). Crystallinity reduction relative to commercial HPC has been proposed based on the lower crystallinity of the underlying bagasse cellulose feedstock, but this has not yet been directly measured for the hydroxypropylated product. Beyond performance, bagasse is an agricurtural residue available at negligible feedstock cost, in contrast to the established market prices of purified wood pulp and cotton linter (US$18–25 per kg) used in commercial HPC manufacturing. Life-cycle assessments of bagasse valorization pathways have similarly reported favorable environmental profiles relative to conventional biomass feedstocks. However, no dedicated techno-economic or life-cycle assessment specific to pharmaceutical-grade HPC production from bagasse has been published, and these potential sustainability and cost advantages therefore remain to be formally validated at industrial scale. Key challenges remain in regulatory acceptance, impurity control, batch-to-batch standardization, and scaling up etherification under pharmaceutical good manufacturing practice (GMP) constraints. Overall, the reviewed literature positions sugarcane bagasse (SCB)-derived HPC as a promising candidate for combining excipient performance with potential sustainability and cost benefits, necessitating targeted process optimization and qualification studies to expedite industrial adoption.

## 1. Introduction

**Figure 1:**
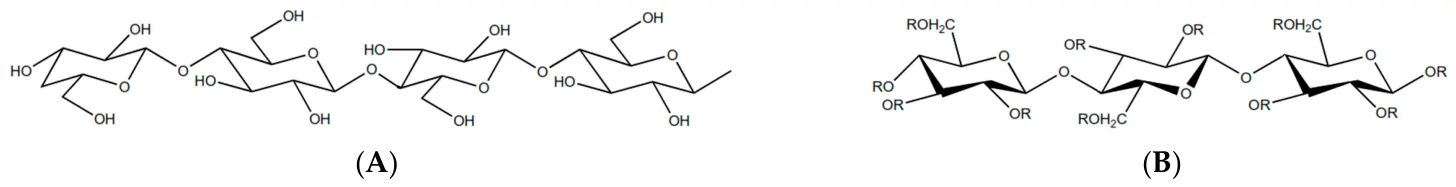
Chemical structure of cellulose (**A**) and hydroxypropyl cellulose (HPC) (**B**), where R=H or –CH_2_CH(OH)CH_3_.

The increasing focus on sustainability and environmental responsibility in pharmaceutical development has intensified interest in biopolymer-based excipients sourced from renewable biomass. This trend spans diverse biomass-derived polymer classes including polysaccharides, lignin, and proteins being explored for drug delivery design, reflecting a broader shift toward renewable feedstocks across pharmaceutical formulation science [1]. Hydroxypropyl cellulose (HPC) (chemical structure shown in Figure 1B) is a non-ionic cellulose ether that has gained widespread acceptance in pharmaceutical formulations due to its biocompatibility, superior film-forming capabilities, thermoplastic characteristics, and functional versatility in immediate- and modified-release oral dosage forms [2,3]. Historically, HPC is produced from high-purity α-cellulose sourced from wood pulp or cotton linter. However, the availability of pharmaceutical excipients is being increasingly evaluated not only in terms of technical performance, but also through a broader supply-risk and resilience perspective [4,5].

Beyond technical performance, the sourcing of pharmaceutical-grade cellulose is increasingly evaluated through supply-chain resiliencee, sustainability governance, and cost-stability lenses. Dependence on globally traded wood pulp or cotton linter exposes manufacturers to risks including geopolitical disruptions, freight volatility, and emerging regulatory pressures such as the EU Deforestation-free Products Regulation (EUDR) [6,7].

Concurrently, commodity price volatility in cotton and woody biomass markets creates procurement uncertainty for excipient manufacturers. In this context, SCB-derived HPC represents a strategically attractive cellulose source that can reduce exposure to theese supply-chain vulnerabilities while supporting circular bioeconomy principles, as shown in Table 1. Feedstock price for wood pulp refers to the raw, traded dissolving-grade pulp commodity (intermediate cellulose feedstock), not the finished HPC excipient. This figure should not be confused with the production cost of finished commercial HPC (US$18–25/kg), discussed in Section 3.16 (Commercial HPC: Regulatory and Economic Considerations); the difference reflects the additional alkalization, etherification, and pharmaceutical-grade purification steps required to convert raw pulp into HPC. Cotton linter feedstock price is not consistently reported across available sources and is therefore marked as requiring verification rather than presented as a point estimate.

Sugarcane bagasse, a fibrous by-product of the sugar industry, represents an abundant yet underutilized resource. Agricultural waste represents a promising resource in this context [13,14]. Furthermore, several agricultural waste products have been utilized as raw materials for the extraction of industrially valuable products, including sugarcane bagasse microcrystalline cellulose (SCBMCC) and nanocrystalline cellulose (CNC), among others. Bagasse is composed of 45−60% cellulose, 20−25% hemicellulose, 18−24% lignin, 1−4% ash, and less than 1% wax (based on dry weight). Annually, a substantial quantity of agricultural waste is gathered worldwide [15]. The production of sugar from 10 wet tonnes of sugarcane generates 3 wet tonnes of sugar fiber waste, which contributes to environmental pollution if disposed of improperly [16,17]. Considering its scale, sugarcane bagasse represents a highly profitable alternative for cellulose extraction with minimal environmental impact. Sugarcane bagasse, characterized by its high cellulose content (40–50%) and relatively low lignin-to-cellulose ratio, presents a promising substrate for the synthesis of cellulose derivatives, particularly hydroxypropyl cellulose (HPC) [18,19]. Cellulose extracted from bagasse can be chemically modified through alkalization and subsequent etherification with propylene oxide to produce HPC. This process allows for the adjustment of physicochemical properties, including degree of substitution (DS) and molar substitution (MS), which influence solubility, swelling capacity, and mechanical behavior in pharmaceutical formulations [3]. The structural parameters are essential in determining the performance of HPC as an excipient, particularly in tablet manufacturing and controlled drug delivery systems.

Although there is a growing interest in cellulose ethers sourced from non-wood biomass, thorough reviews specifically addressing HPC derived from sugarcane bagasse are limited. The existing literature primarily focuses on hydroxypropyl cellulose derived from traditional materials or its applications in non-pharmaceutical sectors, including textile coatings, optics, and food packaging [20,21]. Currently, the pharmaceutical potential of bagasse-based HPC, utilized as a tablet binder with advantageous compaction properties or as a drug delivery matrix that can modulate release kinetics, remains inadequately assessed in a systematic and comparative framework. Preliminary research on lignocellulosic sources, including Betung bamboo, suggests that biomass-derived HPC can attain tablet hardness, friability, and disintegration characteristics similar to those of commercially available HPC grades such as Klucel™ [22]. Moreover, recent technological advancements, including hot-melt extrusion and 3D printing, have enhanced the pharmaceutical applications of HPC, especially in the realms of personalized medicine and intricate drug release profiles [23,24]. Recent studies on in situ gelling systems and thermosensitive hydrogels have underscored the potential of HPC in innovative dosage forms for targeted or injectable drug delivery [25]. These advantages have, to date, been demonstrated exclusively using commercial wood- or cotton-derived HPC; whether they extend to sugarcane bagasse (SCB)-derived HPC remains unverified and is examined as a priority research need in Section 3.17 (Evidence Gaps and Research Needs). This review seeks to address the existing gap by critically assessing the synthesis pathways, physicochemical characterization, and pharmaceutical performance of hydroxypropyl cellulose obtained from sugarcane bagasse. The focus is on its function as a binder in tablet formulations and as a matrix for controlled-release systems, accompanied by a comparative analysis with commercial HPC products. This review synthesizes findings from 106 peer-reviewed publications, providing a targeted and practical perspective on the valorization of sugarcane bagasse in sustainable pharmaceutical manufacturing. This article aims to serve as a resource for researchers and formulation scientists in the development of environmentally friendly and functionally effective excipients derived from agro-industrial residues.

## 2. Methodology

This article presents a structured narrative review, a methodology deliberately chosen over a fully systematic or scoping review design given the early-stage and heterogeneous nature of the evidence base on sugarcane bagasse-derived hydroxypropyl cellulose (SCB-derived HPC) for pharmaceutical applications. A structured narrative review is particularly appropriate when primary studies are scarce, with methodologically diverse protocols, and report outcomes that are not readily comparable across studies. Accordingly, this review incorporates transparent procedures for literature search, study selection, and critical appraisal, informed by key elements of the PRISMA 2020 reporting guidelines and the SANRA principles for narrative reviews, to maximize methodological clarity and reproducibility while retaining the analytical flexibility appropriate for an emerging field.

### 2.1. Search Strategy and Database Selection

A comprehensive literature search was conducted across three bibliographic databases to minimize database-specific bias, including Scopus, Science Direct, PubMed/MEDLINE, and Embase to identify and synthesize pertinent peer-reviewed studies on the synthesis, characterization, and pharmaceutical applications of hydroxypropyl cellulose (HPC), particularly those derived from sugarcane bagasse and other polysaccharides. Search strings combined controlled vocabulary and free-text terms for (i) hydroxypropyl cellulose/HPC, (ii) sugarcane bagasse/bagasse cellulose, and (iii) pharmaceutical excipient/drug delivery terms. A representative Boolean string was “hydroxypropyl cellulose” AND “sugarcane bagasse” OR “bagasse cellulose” OR “sugarcane residue” AND excipient OR pharmaceutical OR “drug delivery” OR “matrix” OR “hydrogel” OR “film”. The selection criteria emphasized original research articles and reviews published in English that specifically focused on the chemical modification of cellulose, the functionality of HPC in drug delivery, and the potential of lignocellulosic biomass, particularly sugarcane bagasse, as a feedstock natural source of pharmaceutical cellulose derivatives. This method established a concentrated and high-quality evidence foundation for the current review.

### 2.2. Screening and Selection

Following the search strategy outlined above, records were identified through database searching. A total of 310 records across the three databases (PubMed: *n* = 2; ScienceDirect: *n* = 304; and Scopus: *n* = 4) were retrieved for initial screening. All retrieved records were imported into a reference management spreadsheet for systematic screening. After excluding non-English publication, conference abstracts, patents, and articles unrelated to cellulose-based excipients based on initial title screening, 120 records were retained for title and abstract review against the inclusion criteria. After the removal of 12 duplicate records, 108 unique records remained and screened. Full text articles for all 108 records were retrieved and assessed for eligibility against the predefined inclusion and exclusion criteria, concentrating on studies. Duplicate entries were removed after initial title-based exclusion (rather than immediately after database retrieval) because duplicate records were more reliably identified once non-relevant records were removed, ensuring that the selected literature is specifically focused on HPC synthesis, characterization, and pharmaceutical applications. The studies were categorized into the following three thematic groups for structured analysis: (a) synthesis and chemical modification of hydroxypropyl cellulose, (b) physicochemical characterization and material properties, and (c) pharmaceutical performance and functionality as an excipient. This classification provides a structured framework for the critical comparison and synthesis of findings across various research dimensions.

### 2.3. Inclusion Criteria

Eligibility criteria were defined a priori to reduce subjectivity. This review encompassed studies that fulfilled the following specified criteria: (1) the synthesis or chemical derivatization of hydroxypropyl cellulose (HPC) focusing on sources from sugarcane bagasse or other lignocellulosic biomass; (2) reported at least one quantifiable output relevant to either material performance (DS, molecular weight/viscosity, crystallinity, and solubility/hydration behavior) or pharmaceutical functionality (tablet binding/compaction metrics, film/mechanical properties, swelling/gel behavior, drug release profiles, and stability); (3) physicochemical characterization with established analytical techniques such as Fourier-transform infrared spectroscopy (FTIR), scanning electron microscopy (SEM), thermogravimetric analysis (TGA), differential scanning calorimetry (DSC), and X-ray diffraction (XRD); (4) pharmaceutical performance parameters were evaluated, including tablet hardness, friability, disintegration time, and drug release behavior; and (5) the findings were published in peer-reviewed scientific journals and are available in full-text English-language versions. These criteria facilitated the selection of studies with methodological rigor, analytical depth, and relevance to the application of HPC as a pharmaceutical excipient.

### 2.4. Exclusion Criteria

Studies were omitted if they did not conform to the pharmaceutical emphasis of this review. Articles were excluded if they focused on non-pharmaceutical applications of hydroxypropyl cellulose (HPC), including its utilization in food packaging, electronics, or the textile industry. Research that concentrated on cellulose derivatives unrelated to HPC was also omitted. Furthermore, review articles, editorials, conference abstracts, and patents were excluded, as the objective was to include solely original research containing primary data. Publications that did not provide adequate methodological detail or clarity in their reported results were excluded to maintain the reliability and reproducibility of the collected evidence.

### 2.5. Data Extraction

Relevant data were systematically extracted from each selected study to enable thematic analysis. For each study, the following parameters were extracted when reported: degree of substitution (DS), molar substitution (MS), crystallinity index (CrI), viscosity grade, tablet hardness/friability/disintegration time, and drug release parameters (e.g., Korsmeyer–Peppas release exponent *n*). The collected key information also included the type of biomass or cellulose source used, as well as detailed parameters concerning HPC synthesis, such as the type of alkaline pretreatment, the etherifying agent employed, and reaction conditions. Characterization methods were documented, focusing on techniques that evaluate thermal stability, surface morphology, and crystallinity, including thermogravimetric analysis (TGA), differential scanning calorimetry (DSC), scanning electron microscopy (SEM), and X-ray diffraction (XRD). The functional performance of HPC as a pharmaceutical excipient was documented, particularly its roles as a tablet binder, film-coating polymer, and controlled-release matrix. Comparative evaluations with commercially available HPC products, including various grades of Klucel™, were documented when available. All extracted data were systematically organized into structured tables and categorized thematically to facilitate a comprehensive narrative synthesis.

### 2.6. Quality Assessment

Although this review adopted a narrative approach instead of a fully systematic approach, this methodological choice was deliberate and appropriate for the scope of the topic. A narrative review is particularly suited for emerging and heterogeneous research areas where the available evidence is limited, methodologically diverse, and cannot be quantitatively pooled through meta-analysis. The field of SCB-derived HPC for pharmaceutical applications represents precisely such a topic: primary studies are scarce, employ diverse synthesis and characterization protocols, and report outcomes that are not readily comparable across studies. A fully systematic review with meta-analytic synthesis would therefore be methodologically premature at this stage of the evidence base [26]. While systematic reviews are considered the gold standard for evaluating well-defined clinical questions with a sufficient body of homogeneous evidence [27], narrative reviews provide the flexibility to integrate evidence across diverse methodologies, trace the conceptual development of a field, and generate hypotheses for future investigation.

### 2.7. Conclusion of Methodology

**Figure 2:**
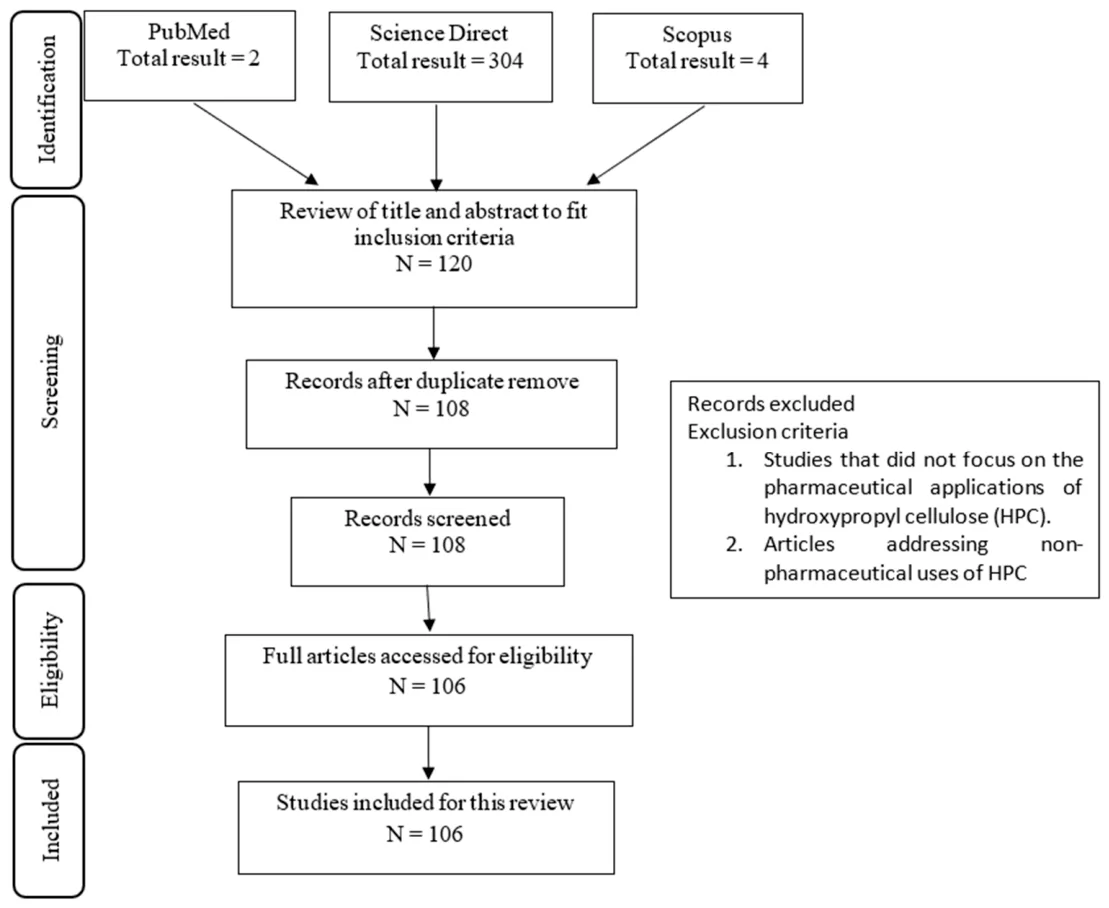
Flow chart showing the literature search.

This review employed a structured and transparent search and selection process, summarized in Figure 2, to identify the high-quality, peer-reviewed literature on SCB-derived HPC. The resulting dataset of 106 studies provides the evidentiary foundation for assessing sugarcane bagasse as a sustainable source of HPC for pharmaceutical applications. Mendeley version 1.19.8 and EndNote 20 were used to organize and manage all studies selected for inclusion in this review. The flow of this selection process is summarized in Figure 2.

## 3. Results

### 3.1. Synthesis of Hydroxypropyl Cellulose (HPC) from Sugarcane Bagasse

#### 3.1.1. Selection of Raw Material and Cellulose Content

Sugarcane bagasse (SCB) is a fibrous byproduct of the sugar milling industry, including roughly 40–50% cellulose, in addition to hemicellulose and lignin [25]. Its comparatively elevated cellulose content and diminished lignin-to-cellulose ratio render it a viable choice for the manufacturing of cellulose derivatives, including hydroxypropyl cellulose (HPC) (structure in Figure 1B). The cellulose content of different plant-based agro-industrial wastes exhibits significant variation, as indicated by literature studies and the data in Table 2. Banana fibers [22] demonstrate the highest cellulose content at 64.04%, with *Ficus macrocarpa* bark [24] at 48.4% and sugarcane bagasse at 42.4% following. While the cellulose content in sugarcane bagasse is lower than that in banana fibers, it remains higher than that found in empty fruit bunches of oil palm [23], which contain merely 36.7% cellulose. This comparison underscores sugarcane bagasse as a viable plant-derived source of cellulose, particularly due to its comparatively low lignin content. Sugarcane bagasse serves as a viable and sustainable alternative for producing cellulose derivatives, including hydroxypropyl cellulose (HPC), reflecting the increasing interest in renewable biomass for value-added applications, whilst bamboo-derived cellulose attains approximately 60–65%, suggesting that sugarcane bagasse is a viable alternative for cellulose alongside these materials [17,21].

Sugarcane bagasse presents multiple advantages as follows: (i) it is plentiful and underutilized within the sugar industry; (ii) it is renewable and environmentally sustainable; and (iii) it possesses an appropriate cellulose profile for derivatization. The application of this approach is consistent with circular economy strategies and has the potential to substantially decrease reliance on wood-based cellulose. Thus, it demonstrates significant potential as a raw material for the sustainable production of HPC [19]. The moisture content of raw sugarcane bagasse significantly influences cellulose yield during the delignification and hydrolysis stages. High water content (>10%) dilutes the alkali concentration, reduces delignification efficiency, and leads to incomplete hemicellulose removal, resulting in lower cellulose recovery. In contrast, pre-dried bagasse (moisture < 8%) improves alkali penetration and enhances cellulose purity. Previous studies reported that reducing the moisture level from 15% to 8% increased α-cellulose yield from 38% to 47% [21,25]. Therefore, controlling the initial water content before pretreatment is critical for achieving consistent cellulose yield and quality suitable for pharmaceutical-grade hydroxypropyl cellulose (HPC) synthesis.

#### 3.1.2. Pretreatment and Isolation of α-Cellulose

**Figure 3:**
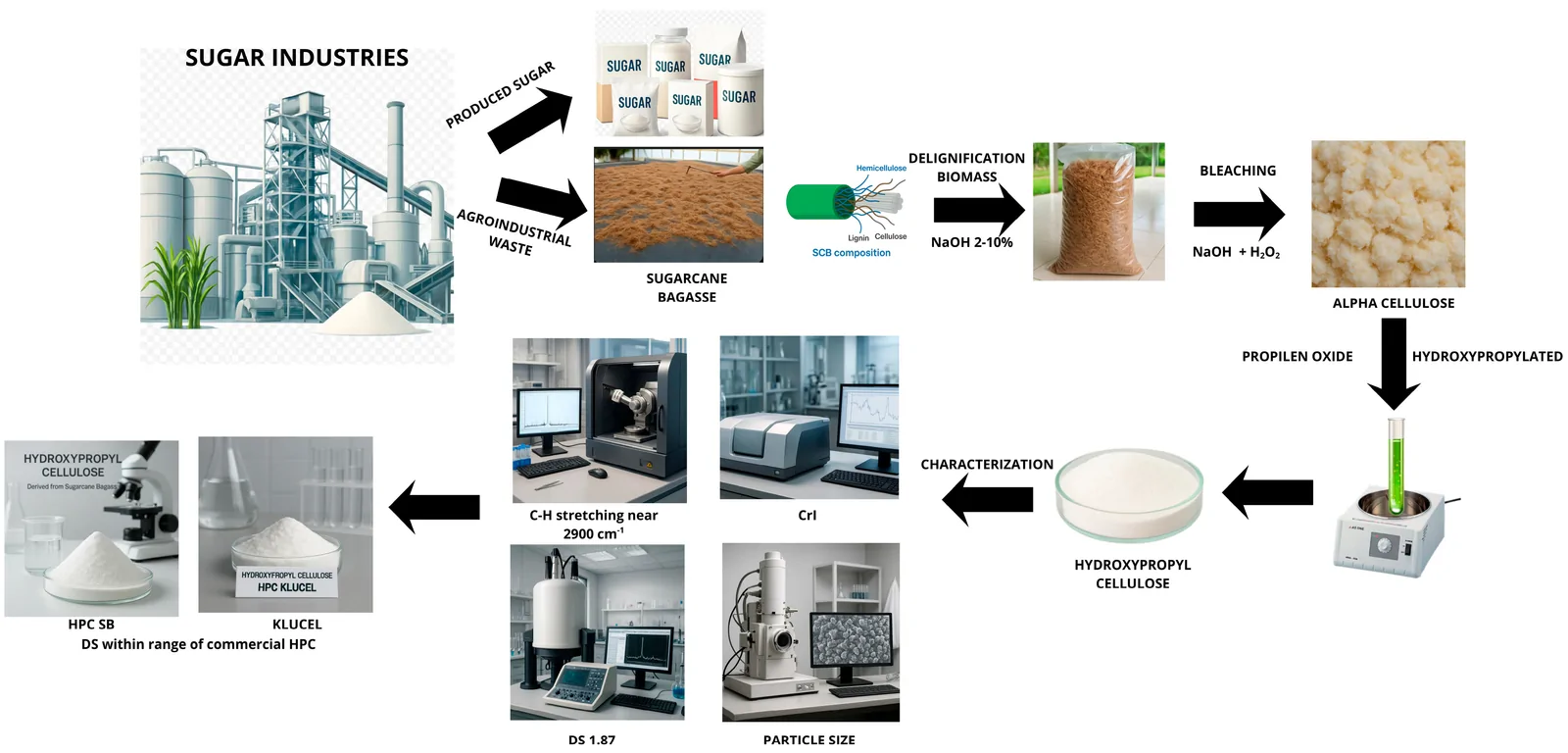
Synthesis flow of hydroxypropyl cellulose (HPC) from sugarcane bagasse.

The transformation of raw sugarcane bagasse (SCB) into α-cellulose necessitates a multi-step pretreatment to eliminate hemicellulose, lignin, ash, and extractives. Alkaline delignification is performed with sodium hydroxide (NaOH) solutions (2–10%) to solubilize lignin and hemicellulose at elevated temperatures (100 °C). Bleaching with sodium chlorite (NaClO_2_), hydrogen peroxide (H_2_O)_2_, or sodium hypochlorite (NaOCl) [31] under acidic circumstances effectively eliminates residual lignin and improves cellulose purity (Figure 3). The neutralization and washing processes utilizing hydrochloric acid (HCl) and deionized water guarantee the elimination of metal ions and the stabilization of the finished product. This procedure yields pure α-cellulose, ranging from white to off-white, with a concentration exceeding 60%, appropriate for subsequent etherification. The yield of α-cellulose from sugarcane bagasse varies between 30% and 50%, depending upon the intensity and efficacy of the pretreatment conditions [17,19]. More recently, response surface modeling (RSM) has been applied to optimize delignification and mercerization parameters for SCB cellulose isolation, demonstrating that systematic process optimization can substantially improve yield and purity beyond what fixed-condition protocols typically achieve [32]. In addition to conventional alkaline delignification, several green chemistry-based techniques have emerged for cellulose isolation from lignocellulosic biomass. These include deep eutectic solvents (DESs) composed of choline chloride and lactic acid, which can effectively dissolve lignin at mild temperatures (90–110 °C) with high recyclability [10]. Ionic liquid pretreatment (e.g., 1-ethyl-3-methylimidazolium acetate) has also been demonstrated to achieve over 90% delignification efficiency while preserving cellulose integrity [26]. Moreover, enzymatic hydrolysis using cellulase-assisted processes provides an eco-friendly alternative to chemical routes, minimizing waste and energy consumption [17]. These approaches align with the principles of green chemistry and contribute to sustainable cellulose production for pharmaceutical-grade excipients. Compared to wood pulp and cotton linters, which are supplied as unpurified, high purity α-cellulose (>90%) requiring minimal further processing, raw SCB contains substantially more non cellulosic impurities (20–25% hemicellulose, 18–24% lignin), necessitating a more elaborate multi step pretreatment (alkaline delignification, bleaching, and neutralization) to reach comparable purity [31].

#### 3.1.3. Alkalization Stage

Following pretreatment, cellulose is subjected to alkalization, where it is treated with NaOH solution (generally 10–25%) in a non-aqueous medium, such as isopropanol. The non-aqueous medium is critical to control cellulose swelling, promote uniform accessibility of the NaOH, and minimize detrimental side-reactions with water that could reduce subsequent etherification efficiency. This process activates hydroxyl groups in the anhydroglucose (AGU) units, resulting in the formation of alkali cellulose and at the same time increasing its reactivity for the subsequent substitution reactions. This step is crucial to ensure efficient etherification or hydroxypropylation in the next step of the process [3]. The same alkalization chemistry applies to wood pulp and cotton linters, typically using higher NaOH concentrations (25–50%) due to their already-high cellulose purity. SCB requires lower NaOH concentrations (10–25%) combined with non-aqueous co-solvent (e.g., isopropanol), to ensure uniform activation despite its greater feedstock variability [3,21].

#### 3.1.4. Etherification (Hydroxypropylation) Process

The alkali cellulose is subsequently reacted with propylene oxide, the etherifying agent, to incorporate hydroxypropyl groups. Etherification is generally conducted under heterogeneous slurry conditions at temperatures ranging from 40 to 60 °C for a duration of 2 to 4 h [21]. Optimal reaction conditions were established using 1.5 M NaOH and 27.82 M propylene oxide per AGU at 55 °C for 3.5 h, resulting in a DS of 1.15, sufficient for pharmaceutical applications. The DS serves as a vital quality indicator, impacting the solubility, film-forming capacity, and viscosity of HPC. The etherification chemistry of propylene oxide with cellulose is in principle identical regardless of the cellulose sources. Commercial HPC (Klucel™), derived from highly uniform wood/cotton cellulose, consistently achieves DS values of between 1.8 and 2.5. To date, only one study has reported the direct synthesis of HPC from sugarcane bagasse that achieves a DS of 1.87 under heterogeneous alkalization-etherification conditions [33]. While SCB’s DS value is comparatively lower and more variable crystallinity is expected, which may influence reactivity and substitution uniformity, this effect has not yet been systematically investigated across multiple SCB-derived HPC synthesis studies, representing an important avenue for future process optimization research.

### 3.2. Factors Affecting Reaction Efficiency

The efficiency of a reaction is affected by factors such as temperature, time, alkali content, and the ratio of propylene oxide [32,34]. Moreover, the structural characteristics of cellulose, especially its crystallinity, affect reactivity [35]. Biomass such as sugarcane bagasse, which often displays lower crystallinity compared to wood pulp, undergoes substitution processes more effectively due to its higher proportion of amorphous regions [36,37].

### 3.3. Optimized Degree of Substitution (DS)

The DS is defined as the average number of hydroxyl groups per unit (AGU) that bear a hydroxypropyl substituent, with a theoretical maximum value of 3. These parameters are critical determinants of the solubility, viscosity, and pharmaceutical application of HPC [33]. The only hydroxypropyl cellulose synthesized directly from sugarcane bagasse reported in the literature to date achieved a DS of 1.87 [33], a value consistent with general HPC–structure–property relationships indicating that DS values exceeding 1.0 are sufficient to confer adequate water solubility and pharmaceutical relevance [3,21]. Broader DS ranges (1.0–2.5) reported across the wider HPC literature largely reflect commercial wood- or cotton-derived grades; whether such variability is similarly attainable from SCB-derived HPC remains to be established through additional synthesis studies. Certain protocols also provide the MS, which considers multiple substituents per AGU and correlates with hydroxypropyl content, typically ranging from 40% to 60%. DS is most commonly determined via combination of ^13^C and ^1^H NMR spectroscopy. From the ^13^C NMR spectrum, the ratio R is defined as the integrated signal intensity of internal methyl carbons (I_2_) relative to terminal methyl carbons (I_1_) of the hydroxypropyl side chains, providing an estimate of the average oxypropylene chain length. Combined with the MS value obtained from ^1^H NMR, DS can be estimated using the relationship [27]:

$$DS=\frac{MS}{1+R}$$

### 3.4. Reaction Parameters

The synthesis efficiency, DS, and yield are significantly influenced by the concentration of NaOH, which typically ranges between 1.0 and 1.5 M [34]. An excess of base enhances activation; however, it may pose a risk of cellulose degradation at higher concentrations [19]. Besides NaOH, the optimal loading of propylene oxide concentration is also important, which may range from 13.91 to 27.82 mol per AGU. Excessive use of propylene oxide may lead to the formation of byproduct. The reaction temperature of 50–60 °C promotes effective etherification and reduces thermal degradation [34]. The optimal reaction time of 2.5–4.0 h ensures an effective balance between reaction completeness and material integrity will be achieved. The solid-to-liquid ratio affects both reactivity and homogeneity, with a common application range between 1:10 and 1:20 (*w*/*v*). Meticulous optimization of these parameters guarantees uniform product quality, satisfactory yield (50–85%), and reproducibility [38].

### 3.5. Physicochemical Characterization of Hydroxypropyl Cellulose (HPC)

The physicochemical characterization of HPC is crucial for assessing its suitability as a pharmaceutical excipient, especially as tablet binder and drug delivery matrix. This section describes the analytical techniques and parameters employed to evaluate the structure, functional properties, and quality of HPC derived from sugarcane bagasse.

### 3.6. Molar Substitution (MS) and Substitution Pattern

Molar substitution (MS) is defined as the average number of moles propylene oxide that have reacted per AGU, including additional hydroxypropyl groups introduced via chain extension of previously substituted hydroxyls, and hence, MS values may exceed 3. MS parameters are critical determinants for solubility, viscosity, thermal properties, and biofunctionality of HPC [38]. While most hydroxypropylation routes follow a similar sequence (by alkalization and etherification with propylene oxide), the pharmaceutical usefulness of HPC is ultimately dictated by how process variables control substitution chemistry and chain architecture [34]. Alkali concentration and alkalization conditions govern cellulose activation and accessibility of hydroxyl sites, thereby influencing the attainable substitution level and its distribution [39]. The ratio of propylene oxide (PO) and the reaction temperature and time are the primary determinants of the extent of etherification, influencing the balance between the targeted DS and the MS profile [3]. Notably, the existing literature indicates that both the average MS/DS and the heterogeneity or substitution pattern can significantly impact the physicochemical behavior, even when technical specifications are considered (Table 3) [28].

Increasing the degree of hydroxypropyl cellulose substitution disrupts both intra- and intermolecular hydrogen bonding within cellulose, thereby enhancing solvent–polymer interactions and promoting greater aqueous compatibility. In modified cellulose, solubility and rheological properties are reported to be contingent upon the MS, the distribution of substituents, and the molecular weight. Regioselective or heterogeneous hydroxypropylation can achieve water solubility at MS values around 0.8 [40]. Broader datasets reveal a significant trend wherein increased hydroxypropyl substitution enhances solubilization and modifies rheological characteristics. Given that viscosity grade in aqueous media serves as a functional indicator of chain entanglement and hydration state [3], the DS/MS and substitution pattern become practical parameters for adjusting wetting, gel formation, and diffusion pathways in dosage forms [29]. Proton NMR spectroscopy is the most precise technique for determining molecular structure. The samples undergo hydrolysis in deuterated chloric acid (DCl), and the integration of characteristic methyl and anomeric proton signals is employed to calculate MS values [41]. Complementary gas chromatography (GC) analysis, utilizing 2-iodopropane as a derivative, is employed alongside correction equations calibrated against NMR results to improve accuracy [41]. The arrangement of substituents along the cellulose chain is defined by enzymatic hydrolysis via endo-1,4-β-D-glucanase, followed by the determination of released reducing sugars (RSs) via the DNS assay [28]. Elevated RS levels signify increased heterogeneity and a higher presence of low-substituted regions, potentially affecting hydration, film formation, and drug release characteristics. Based on the available literature [27,30], the MS of HPC is most reliably determined via ^1^H NMR spectroscopy, by comparing the integrated signal intensity of the methyl protons of the hydroxypropyl substituents ((H9, δ ≈ 1.0–1.2 ppm) relative to the anomeric proton of the AGU backbone (H1, δ ≈ 4.2–4.6 ppm) [23]; here are equations that can be used to calculate the MS values:

Equation (1) [29]:

(1) $$MS=\frac{I_{9}}{3\times I_{1}}$$

where I_9_ and I_1_ denote the integrated peak areas of the hydroxypropyl methyl and anomeric proton signals, respectively. This NMR-based method is the most widely validated approach for MS determination in HPC characterization studies.

**Table 3:** Influence of processing parameters on the structural and functional properties of hydroxypropyl cellulose for tablet performance and drug release.

Processing Parameter	Effect on DS/MS	Effect on Viscosity	Impact on Tablet Performance	Impact on Drug Release	References
Alkali concentration (NaOH)	↑ Alkali → ↑ accessibility of hydroxyl groups → positive correlation to DS/MS (up to optimal level)	↑ DS/MS → positive correlation to viscosity (higher substitution = thicker polymer in solution)	↑ Binding strength and compressibility (positive) due to more uniform substitution and chain mobility	↓ Drug release (more sustained) due to stronger matrix formation	[22,34,38]
Propylene oxide (PO) ratio	↑ PO ratio → positive correlation to DS/MS (more hydroxypropyl groups added)	↑ Viscosity (positive) with higher substitution, though plateau at very high PO	↑ Tablet mechanical strength (positive) via enhanced polymer–polymer interactions	↓ Release rate (matrix densifies)	[18,22,42,43,44]
Solvent system (aqueous vs. aqueous–organic)	Mixed solvents (e.g., DMSO systems) → more uniform substitution distribution → positive correlation to effective DS/MS	Uniform substitution → positive effect on consistent viscosity and reproducibility	Better granulation uniformity → ↑ tablet robustness	More predictable release profiles (positive consistency)	[42,45,46]

### 3.7. Crystallinity

X-ray diffraction (XRD) is commonly used to evaluate the crystallinity of HPC, a property that influences its solubility and processability. HPC typically exhibits two characteristic diffraction peaks at approximately 8° and 20° 2θ, consistent with a partially crystalline structure. Across the general HPC literature, the crystallinity index (CI) of HPC (approximately 71%) is reported to be lower than that of microcrystalline cellulose (MCC, approximately 87%), suggesting comparatively greater solubility and formulation compatibility for HPC. It should be noted that XRD characterization specific to SCB-derived HPC has not yet been reported in the primary literature; the values above are derived from studies of commercial HPC and presented here for general context rather than as direct evidence for SCB-derived HPC. Variations in the crystallinity index (CrI) and hydration rate are pertinent to formulation, as they influence water accessibility, polymer swelling/gel formation, and ultimately the mechanical integrity and disintegration/release characteristics of dosage forms [32]. Thus, a feedstock/process route that yields CrI and/or expedited hydration (as delineated in Table 1) can influence (i) the initiation and intensity of gel-layer formation and (ii) water infiltration into the compact, two mechanisms that directly govern both disintegration and sustained-release efficacy. From a tablet-manufacturing perspective, the significance of Table 3 lies in the mechanism of how structural changes affect the compressibility and bonding mechanisms. HPC is extensively utilized as a binder due to its mechanical and thermal properties, as well as its molecular characteristics, which facilitate particle bonding during compaction. Consequently, any alterations that affect chain accessibility and hydration behavior, such as changes in CrI-related water–polymer interactions, are anticipated to impact the formation of tensile strength and tablet integrity. Thus, the CrI/hydration measures in Table 3 should be viewed as upstream markers for downstream tablet ability rather than as independent material descriptors [33]. Crystallinity index (CI) is calculated using the Segal method [25]:

$$CI=\frac{I_{002}\;{-I}_{AM}}{I_{002}}\times 100$$

where I_002_ and I_AM_ represent crystalline and amorphous peak intensities, respectively.

### 3.8. Hydration Rate

The hydration kinetics of HPC are conventionally assessed by monitoring the dynamic viscosity of 1% (*w*/*v*) HPC solutions at defined time intervals (e.g., 1 and 24 h) using a rotational rheometer. HPC grades with enhanced water affinity exhibit faster hydration, a property considered advantageous for immediate release tablet formulations, as polymer hydration rate governs the formation of a protective gel layer and the subsequent balance between diffusion and erosion controlled drug release. To date, no hydration kinetics data specific to SCB-derived HPC have been reported, and this parameter is therefore identified as a priority characterization gap for future work [32].

### 3.9. Rheological Behavior

HPC solutions, in general, exhibit shear-thinning behavior characteristic of polymeric solutions, well described by the Herschel–Bulkley model. These rheological characteristics have been documented for commercial HPC grades, corresponding rheological data for SCB-derived HPC have not yet been reported and remain an open research question. Rheological profiles are critical for comprehending processability in granulation and tablet compression. In a rheological study of 17 HPC-based filament formulations for material-extrusion 3D printing, incorporating various drug loads, co-polymers, and disintegrants, complex viscosity decreased with increasing temperature across all formulations, with shear-thinning behavior observed throughout (slope range −0.28 to −0.74); notably shear rate exerted a greater influence on viscosity than temperature did [34]. These characteristics have also made it well-suited as the primary polymer in 3D printing filaments for drug products, both for immediate and controlled release [34]. In solid dosage forms, the DS/MS viscosity directly correlates with essential performance characteristics. In low-substituted hydroxypropyl cellulose (HPC) variants, the concentration of hydroxypropyl groups significantly influences water absorption, swelling pressure, and tablet disintegration times. This suggests that the DS alters the wicking-swelling equilibrium, which governs compact disintegration. In hydrophilic matrices, increased polymer hydration and viscosity generally promote the formation of a more viscous gel layer, potentially hindering drug diffusion and erosion. Conversely, reduced DS/MS should be considered not only as chemical descriptors but also as formulation-level factors that affect (i) interparticulate bonding and compressibility, (ii) disintegration kinetics through water absorption and swelling efficiency, and (iii) drug release regulation via gel strength and diffusion resistance [35]. This remains the only systematic rheological dataset identified for HPC formulations in this review; broader rheological comparison across multiple HPC sources and grades has not yet been reported, representing a further characterization gap consistent with Section 3.17.

### 3.10. Fourier-Transform Infrared (FTIR) Spectroscopy

Fourier-transform infrared (FTIR) spectroscopy, typically performed using the KBr pellet method, is used to confirm the structural identity of HPC and its precursor cellulose. According to Ohwoavworhua [36], α-cellulose derived from agricultural biomass exhibits characteristic absorption bands at approximately 3398 cm^−1^ (O–H stretching), 1060 cm^−1^ (asymmetric C–O–C bridge), and 890 cm^−1^ (β-glycosidic bond). For HPC synthesized directly from sugarcane bagasse pulp. Abou-Zeid et al. (2021) [33] reported O–H stretching at 3390 cm^−1^, C–H stretching of the methylene and hydroxypropyl methyl groups at 2892 and 1420 cm^−1^, and a C–O–C band at 1070 cm^−1^, with the emergence of a distinct double absorption band between 3000 and 2800 cm^−1^ providing direct spectroscopic evidence of successful hydroxypropylation. This remains the only FTIR dataset reported for SCB-derived HPC in the literature to date.

### 3.11. Scanning Electron Microscopy (SEM) Analysis

Scanning electron microscopy (SEM) is widely used to characterize the surface morphology of HPC, typically following gold/palladium sputter-coating and imaging under high-vacuum conditions using a secondary electron detector [27,38]. Baron et al. (2023) [47] characterized the surface morphology of commercial HPC by SEM and reported a distinctive cauliflower-like surface structure in unmodified HPC films, attributed to chain aggregation within crystalline domains during solidification. Following chemical oxidation, this structured morphology was disrupted, with the formation of amorphous regions partially replacing the well-defined domains a finding broadly consistent with the accompanying reduction in crystallinity index observed by XRD for the same sample [27]. It should be noted that this morphological dataset derives from a commercial HPC sample of unspecified cellulose source subjected to post-synthesis chemical oxidation, not from SCB-derived HPC whether for unmodified material or in comparison to commercial grades have been reported in the primary literature, to date representing a further characterization gap consistent with Section 3.17. Sample preparation for SEM typically involves solvent-casting into films, cryo-fracturing for cross-sectional imaging, and high-vacuum imaging at low accelerating voltage (~5 kV) [27].

### 3.12. Evaluation of HPC in Tablet Applications and Controlled Drug Delivery Matrix

HPC is a multifunctional excipient extensively investigated for application in pharmaceutical tablets, notably as a binder, film-former, disintegrant, and matrix-forming polymer for controlled drug release. The compound’s solubility in aqueous and organic solvents, along with its favorable compressibility and thermoplastic properties, renders it appropriate for traditional tableting and innovative techniques like hot melt extrusion (HME) and 3D printing. Therefore, throughout the following sections, evidence is explicitly classified as either (i) experimentally established for SCB-derived HPC, (ii) a theoretical expectation based on general HPC structure–property relationships (applicable across cellulose sources due to a shared chemical backbone), or (iii) an extrapolation from performance data on commercial HPC (wood-/cotton-derived), pending direct validation for SCB-derived HPC. A dedicated discussion of these gaps is provided in Section 3.17, Evidence Gaps and Research Needs.

### 3.13. Tablet Binder and Disintegrant

HPC in low-viscosity grades (e.g., Klucel™ LF, EF, and ELF) is frequently utilized as a binder in immediate-release tablets [38,48]. Its high solubility in water facilitates rapid disintegration and dispersion upon contact with gastrointestinal fluids [49]. According to data from Ashland Inc. (Wilmington, DE, USA), Klucel™ LF (Mw ~95,000) provides effective binding and rapid dispersion while maintaining tablet hardness [39]. However, it should be noted that this binder performance has been characterized for commercial wood-/cotton-derived HPC, and to the best of authors’ knowledge, no equivalent binder performance data for SCB-derived HPC have been reported in the literature.

Based on general structure–property relationships established for HPC regardless of cellulose source, the critical determinants of binding efficacy include the DS and molecular weight, which collectively influence the solution viscosity and polymer chain entanglement. Higher-viscosity hydroxypropyl cellulose (HPC) grades frequently exhibit enhanced binding strength due to superior plastic deformation and an increased contact area among particles [48,50,51]. The non-ionic nature of HPC minimizes electrostatic repulsion, thereby improving cohesive bonding among various active pharmaceutical ingredients (APIs). The affinity for moisture and ability to form films facilitate the formation of solid bridges during drying, thereby reinforcing the mechanical integrity of the tablets [52]. The structure–property relationships elucidate the consistent performance of HPC and common pharmaceutical polymers provided that comparable DS/MS and viscosity profiles are achieved during synthesis; however, this remains to be experimentally confirmed in direct tablet formulation studies (Table 4).

Recent studies on polysaccharides derived from plants, such as an apple [3], Ficus vasta Gum [41], Cassava [42], jute (*Corchorus olitorius*) [43], and Taro (*Colocasia esculenta*) [44], have consistently demonstrated that the binder performance is influenced by a complex interplay of polymer structure, physicochemical properties, and processing conditions. Viscosity, particle size, and availability of functional groups directly affect granule cohesiveness, tablet mechanical strength, and drug release characteristics. While these studies do not involve HPC directly, they offer an analogous conceptual framework for understanding how DS, viscosity, and particle size may govern binder performance in SCB-derived HPC, a mechanistic parallel that nevertheless requires experimental confirmation through dedicated tablet formulation studies using SCB-derived HPC.

### 3.14. Controlled-Release Matrix Former

High-viscosity grades of HPC (e.g., Klucel™ MF, HF) can be used in tablets with sustained-release properties. They form gel layers upon hydration that modulate drug diffusion. For example, Wilson et al. (2017) [23] demonstrated that hydroxypropyl cellulose (HPC)-based matrices fabricated through hot-melt extrusion (HME) can sustain consistent drug release over 8–12 h, contingent upon drug load and polymer composition. This prolonged drug release is advantageous for maintaining stable drug levels in the body over an extended period, thereby enhancing therapeutic efficacy and reducing dosing frequency. This HME-based release performance was demonstrated using commercial wood-derived HPC grades (Klucel™); whether SCB-derived HPC exhibits comparable melt rheology, extrusion processability, and drug release kinetics in HME systems remains unverified and constitutes an important research gap. In other words, HPC helps control the rate at which drug is released into the body, which is important to achieve more controlled therapeutic effects in drug formulations [48].

In addition to its role in sustained-release matrices, low-substituted HPC has been utilized as a tablet film forming, offering benefits such as taste masking, protection against moisture, and improved mechanical strength [38]. Furthermore, HPC-based formulations have shown resistance to dose dumping [53], even in hydroalcoholic environments, as demonstrated through dissolution testing using USP Apparatus II under varying paddle speeds and ethanol concentrations. This property is particularly advantageous for patient safety, especially in populations that may consume alcohol. These dose dumping resistance characteristics have been reported for commercial HPC grades, and analogous alcohol-resistance data for SCB-derived HPC have not been published.

The drug release kinetics of HPC-based systems are often best described by the Korsmeyer–Peppas model, with most formulations exhibiting non-Fickian (anomalous) transport. This indicates a combined mechanism involving both polymer swelling and drug diffusion. Calculated release exponent (*n*) values typically range from 0.5 to 0.8, consistent with diffusion predominantly governed by the hydrated gel layer [54,55]. These kinetic parameters are expected to be broadly applicable to SCB-derived HPC matrices of comparable viscosity and DS, given the shared cellulose backbone chemistry. However, the specific *k* (rate constant) and *n* (release exponent) values for SCB-derived HPC matrices have not been experimentally determined.

Swelling studies further support HPC’s functionality in sustained-release formulations. Increased HPC content enhances the swelling index, promoting water uptake and extending the drug release duration [56]. In interpenetrating polymer network (IPN) hydrogels incorporating chitosan [57], the addition of HPC not only increased matrix rigidity but also improved the controlled release of model drugs such as chlorothiazide and amoxicillin [54]. These IPN hydrogel studies were conducted using commercial grade HPC, but analogous IPN systems incorporating SCB-derived HPC have not yet been investigated. However, to the best of authors’ knowledge, no published studies to date have evaluated SCB-derived HPC in HME 3D-printing applications, given the sensitivity of filament extrusion to melt viscosity and thermal stability [24]. Hence, the suitability of SCB-derived HPC for this technique is a key open question requiring dedicated experimental investigation [48]. Table 5 synthesizes the structural parameters, processing methods, and corresponding drug release behaviors reported across the HPC literature discussed in this section, illustrating how viscosity grade and DS govern processability and in turn drug release kinetics. These findings underscore the need for systematic evaluation of this material across the same parameter space.

While dedicated drug-release data for SCB-derived HPC remain absent from the literature, recent work confirms that unmodified cellulose extracted from other agricultural residues (rice husk and orange peel) can function as an effective controlled-release matrix for a model drug (ibuprofen), achieving near-zero-order release kinetics in multilayer tablet formulations and performing comparably to commercial cellulose in physicochemical characterization [64]. This supports the broader premise that agro-waste-derived cellulosic materials are functionally viable as release-modulating excipients, even though the specific hydroxypropylated SCB system evaluated in this review has not yet been tested in an analogous release study. In the pharmaceutical industry, hydroxypropyl cellulose (HPC) is widely used as a multifunctional excipient in solid dosage forms, film coatings, and controlled-release matrices. Major commercial grades, such as Klucel™ (Ashland Inc.), are primarily manufactured from high-purity wood or cotton-derived cellulose. These materials are favored for their consistent viscosity and substitution patterns [39]. Transitioning to sugarcane bagasse-derived HPC poses regulatory and manufacturing challenges, including batch-to-batch variability in cellulose purity, limited pharmacopeial standards for non-wood sources, and validation requirements for excipient equivalence under ICH Q6A guidelines [3,18]. Despite these barriers, preliminary techno-economic indications suggest the possibility of lower feedstock costs from adopting bagasse as an alternative source; however, industrial-scale feasibility including the magnitude of any cost reduction remains to be validated through comprehensive TEA (Techno-Economic Analysis) and LCA (Life Cycle Assessment) studies. The production cost of HPC is heavily influenced by the cellulose feedstock and purification steps. Conventional wood-derived HPC typically costs US $18–22 per kg, while cotton-based sources reach US $25 per kg due to higher processing and purification costs [49]. Direct cost data for pharmaceutical-grade HPC derived from sugarcane bagasse have not yet been published. As an indirect reference point, techno-economic assessments of bagasse based cellulose production (not HPC and not pharmaceutical grade) report 30–40% lower production costs than wood pulp in a Latin American context [50]. Whether this cost advantage persists once the additional alkalization, etherification, and pharmaceutical-grade purification steps required to convert cellulose into HPC are accounted for has not been established. Hence, to extend this estimate to SCB-derived HPC would require a dedicated TEA rather than direct extrapolation from the available cellulose-production literature.

In cellulose-based processing methodologies, techno-economic analysis (TEA) and life cycle assessment (LCA) studies consistently highlight the critical importance of yield and consumption of chemicals and utilities. For hydroxypropyl cellulose (HPC) derived from bagasse, the primary cost determinants anticipated to influence the minimum selling price include: (i) the yield and purity requirements of cellulose isolation, which dictate the intensity of chemical use and the complexity of unit operations; (ii) the consumption of etherification reagents, particularly sodium hydroxide (NaOH) for alkalization and propylene oxide for hydroxypropylation; (iii) the use of solvents and water, along with the associated recovery and wastewater treatment demands; (iv) the thermal energy required for drying and finishing processes; and (v) the requirements for pharmaceutical-grade quality assurance, including specification testing, impurity control, and batch-to-batch consistency. Consequently, any cost advantage derived from low-cost residue feedstocks is dependent on achieving competitive yields and efficient chemical and solvent recovery at scale [51].

Table 6 can be used to elucidate the connection between technical and economic rationales. For instance, pathways characterized by reduced crystallinity and enhanced hydration may diminish the need for extreme processing conditions to achieve the desired functionality. Conversely, more severe alkalization/etherification conditions, which increase reagent consumption, may heighten purification demands and operational costs. Economic feasibility in this context is therefore best understood as a trade-off between process intensity (involving chemicals, energy, and effluent treatment) versus the performance achieved (DS/MS/viscosity, hydration behavior, and downstream dosage form efficiency) [32]. However, the extent of cost improvements is contingent upon factors such as process yield, recovery of chemicals and solvents, utility requirements, and burdens associated with pharmaceutical-grade purification and quality. Consequently, quantitative cost assessments necessitate a dedicated techno-economic analysis (TEA) based on mass and energy balances that are realistic for industrial applications [51].

### 3.15. The Application of HPC in Pharmaceutical Research

**Figure 4:**
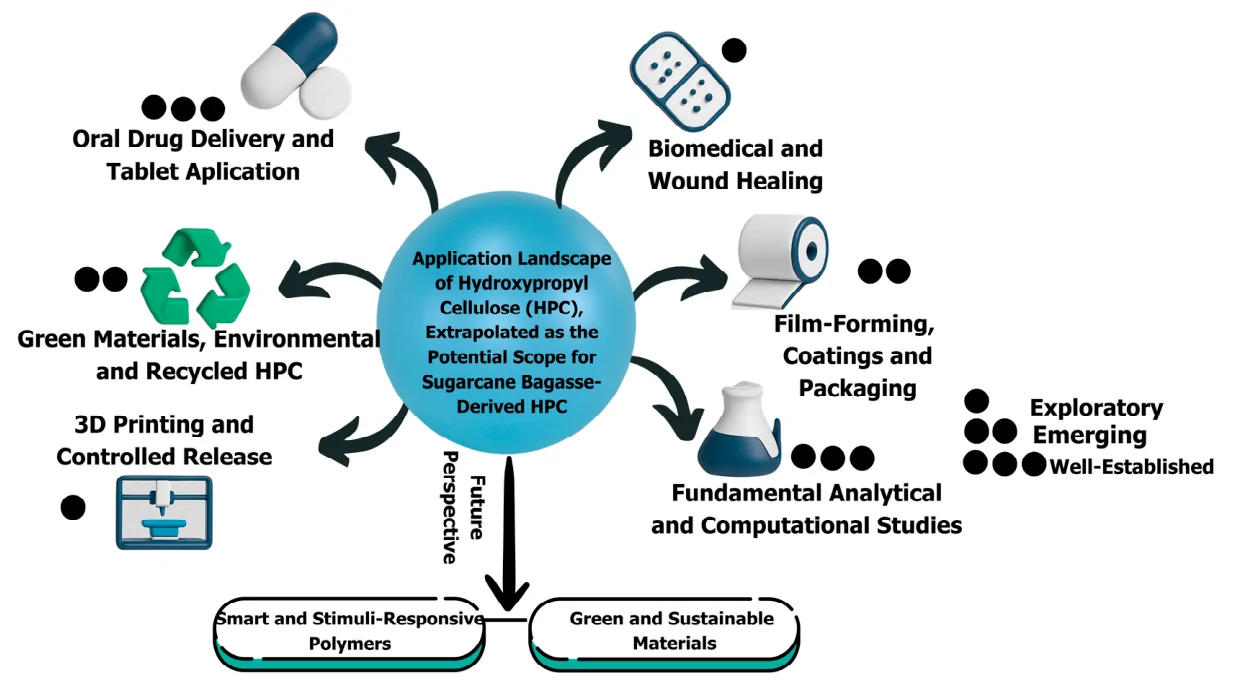
Application of SCB-derived HPC in pharmaceutical research. Dots indicate the relative maturity of underlying literature for each application area: ● Exploratory, ●● Emerging, ●●● Well-Established (See Section 3.15 for definitions).

HPC plays many roles in pharmaceutical and biomedical applications [38]. While studies focusing specifically on SCB-derived HPC are emerging, the established functions of commercial HPC have defined the potential application landscape for this sustainable alternative. We have classified the existing applications reported in the literature into six functional categories to better understand this scope (Figure 4). These categories are included in Figure 4 and Table 7 and are as follows: (1) Oral Drug Delivery and Tablet Applications, (2) 3D Printing and Controlled Release Systems, (3) Biomedical and Wound Healing Applications, (4) Film-Forming, Coatings and Packaging, (5) Green Materials, Environmental and Recycled HPC, and (6) Fundamental, Analytical, and Computational Studies.

The relative maturity ratings shown in Figure 4 reflect the volume and consistency of studies on hydroxypropyl cellulose in general predominantly commercial wood- or cotton-derived grades rather than on SCB-derived HPC specifically, for which direct evidence remains confined to a single synthesis study [33] and is otherwise absent from the pharmaceutical-performance literature reviewed in Table 6. Dot ratings reflect the maturity of general HPC literature (predominantly commercial wood-/cotton-derived HPC) in each application area, not the maturity of SCB-derived HPC specially. Of the 115 studies compiled in Table 7, only one directly involves sugarcane bagasse-derived material; the remaining categorization represents the potential application scope that SCB-derived HPC could occupy if its structure–property equivalence to commercial HPC is experimentally confirmed. For Well-Established categories, application areas are supported by a substantial number of studies with consistent findings and demonstrated functional relevance. Emerging indicators pertain to application areas with a growing body of research showing promising results, albeit with limited comparative or long-term validation. Exploratory indicators refer to application areas investigated in a limited number of preliminary or proof-of-concept studies, where the evidence remains at an early stage. Furthermore, each publication was specifically categorized based on the journal title, drug involved, formulation type, functional purpose, and evaluation methods employed.

### 3.16. Regulatory and Pharmacopeial Perspectives for SCB-Derived HPC

Hydroxypropyl cellulose (HPC) is a recognized pharmaceutical excipient; however, its regulatory acceptance is currently dependent on compendial monographs derived from wood or cotton-based α-cellulose. Prominent pharmacopeias, including the United States Pharmacopeia–National Formulary (USP–NF), European Pharmacopeia (Ph Eur.) The Japanese Pharmacopeia (JP), specify quality attributes such as DS, viscosity, residue on ignition, heavy metals, and microbial limits but do not explicitly differentiate the cellulose source. The introduction of sugarcane bagasse as an alternative cellulose source raises questions regarding excipient equivalence rather than innovation, requiring manufacturers to demonstrate that SCB-derived HPC consistently meets the established compendial standards [150] and does not introduce new impurity risks. Regulatory frameworks, such as ICH Q6A, mandate that changes in raw material sources be supported by comparability data, including physicochemical attributes, impurity profiles, and functional performance in dosage forms. From a quality perspective, non-wood biomass presents additional challenges concerning trace metals, agricultural residues and batch variability [31,151]. Therefore, comprehensive purification methods, validated analytical controls, and pharmaceutical-grade good manufacturing practice (GMP) manufacturing are essential for regulatory approval WHO TRS No. 1060, Annex 3 (2025). A detailed summary of these regulatory and pharmacopeial challenges is provided in Table 8. From a regulatory standpoint, the primary challenge for SCB-derived HPC lies not in the chemical modification itself but in ensuring source traceability, batch consistency, and pharmacopeial compliance [152].

### 3.17. Evidence Gaps and Research Needs

In addition to the pharmaceutical performance gaps discussed above, structural and morphological characterization of SCB-derived HPC remains markedly limited: XRD-based crystallinity, hydration kinetics, rheological profiling, and SEM-based morphological analysis have not yet been reported for SCB-derived HPC, with FTIR being the only characterization technique for which direct SCB-derived HPC data currently exist [33]. While the synthesis and physicochemical characterization of SCB-derived HPC including DS, MS, crystallinity, thermal stability, and hydration behavior have been directly demonstrated in several primary studies reviewed here, pharmaceutical performance data specific to SCB-derived HPC remain scarce. The majority of evidence regarding binder efficacy, controlled-release kinetics, swelling behavior, dose dumping resistance, and suitable for HME and 3D printing applications has been extrapolated from studies on commercial wood- or cotton-derived HPC grades (e.g., Klucel™), based on the reasonable assumption that comparable DS/MS and viscosity profiles will yield comparable functional performance. However, this assumption has not yet been directly validated through systematic comparative formulation studies.

Key research needs identified through this review include: (1) direct comparative binder performance studies—including tablet hardness, friability, and disintegration time—using SCB-derived HPC at matched DS/MS and viscosity relative to commercial HPC grades; (2) dissolution and drug release studies on SCB-derived HPC matrix tablets, including Korsmeyer–Peppas model fitting and comparison of release exponent *n* values; (3) feasibility studies of SCB-derived HPC as a carrier in HME and FDM 3D printing processes, including melt rheology and filament characterization; (4) swelling behavior and IPN hydrogel studies incorporating SCB-derived HPC; and (5) batch-to-batch consistency and pharmacopeial compliance studies under ICH Q6A guidelines. Addressing these evidence gaps is essential before SCB-derived HPC can be positioned as a verified equivalent to commercial grades for pharmaceutical applications.

### 3.18. Future Perspectives

The transition of hydroxypropyl cellulose (HPC) derived from sugarcane bagasse from laboratory research to industrial pharmaceutical applications necessitates coordinated advancements in process engineering, quality assurance, and regulatory compliance. From a manufacturing standpoint, scalability is contingent upon the efficiency of cellulose isolation, recovery of solvents and reagents during etherification, and ability to maintain a consistent DS and viscosity across large production batches. Continuous or semi-continuous processing methods, along with closed-loop solvent recovery, are expected to be crucial for achieving economic viability at a large scale.

Future regulatory efforts should prioritize rigorous comparability studies between bagasse-derived and commercially available HPC grades, focusing on their functional performance in tablets, films, and controlled-release matrices. Establishing excipient equivalence within the ICH and pharmacopeial guidelines is a critical step for broader industrial implementation.

Sustainability considerations are anticipated to increase interest in residue-derived cellulose ethers. As pharmaceutical supply chains increasingly incorporate life cycle assessment (LCA) and deforestation-free sourcing standards, SCB-derived HPC offers significant potential to align excipient functionality with measurable environmental benefits. Future research that integrates techno-economic assessment (TEA) and cradle-to-gate life cycle assessment (LCA) in realistic industrial settings will be essential for quantifying these benefits and informing investment decisions.

Specifically, such an LCA would require explicit definition of: (i) the system boundary—likely cradle to gate, encompassing bagasse collection, delignification, bleaching, and hydroxypropylation, but excluding downstream tablet manufacturing unless a cradle-to-grave scope is adopted; (ii) the functional unit most appropriately 1 kg of pharmaceutical grade HPC meeting compendial specifications, to allow direct comparison with existing LCS data for wood- and cotton-derived HPC; and (iii) allocation assumptions for bagasse as a co-product of sugar and production, since attributing environmental burden between sugar and bagasse outputs significantly affects the calculated footprint of bagasse-derived pathway. None of these parameters have yet been defined in the literature for SCB-derived HPC, underscoring that current sustainability claims remain qualitative rather than quantitatively validated.

## 4. Conclusions

This review has examined the production, characterization, and pharmaceutical application of hydroxypropyl cellulose (HPC) derived from sugarcane bagasse (SCB), a widely available agro-industrial residue. The synthesis pathway from SCB follows the same fundamental chemistry as that used for commercial wood- and cotton-derived HPC—pretreatment to isolate α-cellulose, alkalization, and etherification with propylene oxide—although SCB requires a more elaborate pretreatment due to its higher hemicellulose and lignin content. Despite this additional processing step, the limited data-available evidence indicates that SCB-derived HPC can achieve DS value 1.87 reported in single independent studies within the range reported for commercial HPC grades (DS 1.8–2.5), indicating chemical feasibility of the synthesis route.

However, this review also identified important evidence gaps. Much of the functional performance data (particularly regarding tablet binding, controlled-release behavior, and hot-melt extrusion processability) has been experimentally validated only for commercial wood-/cotton-derived HPC, while direct evidence for SCB-derived HPC in finished dosage forms remains limited. Closing this gap through dedicated tablet formulation and drug-release studies using SCB-derived HPC is therefore a priority for future research.

Beyond its technical performance, SCB-derived HPC offers a compelling sustainability proposition as follows: it valorizes an abundant agricultural byproduct, reduces dependence on wood and cotton feedstocks, and aligns with the pharmaceutical industry’s growing emphasis on circular and bio-based excipient sourcing. Taken together, the evidence reviewed here positions SCB-derived HPC as a scientifically promising and environmentally favorable alternative excipient, contingent upon further translational research bridging laboratory-scale synthesis and validated pharmaceutical performance.

## Figures and Tables

**Table 1:** Comparative overview of primary cellulose feedstocks considered for industrial-grade cellulose production.

Cellulose Source	Global Annual Availability	α-Cellulose Content	Indicative Feedstock Price	Key Sustainability Considerations	Ref
Wood pulp (virgin softwood/hardwood)	~190 million tonnes/year	90–95%	~US$0.6/kg (dissolving-grade export price, 2024)	Reliant on forestry /plantation land, exposure to deforestation linked regulation	[8]
Cotton linter	~1.6 million tonnes/year (byproduct of ~24.7 million tones raw cotton, 2023)	98–99%	Not consistently reported (needs verification)	Co-product of water and pesticide- intensive cotton cultivation	[9,10]
Sugarcane bagasse	~490–600 million tonnes/year (estimates vary by source/year)	40–60% (range across cited studies; see Section 3.1.1)	Negligible/near zero (agricultural residue, no dedicated cultivation)	Agro-industrial residue, no additional land use, currently mostly combusted for mill energy	[11,12]

**Table 2:** Chemical compositions of diverse agro-industrial waste.

Source	Cellulose	Hemicellulose	Lignin	Ref
Banana Fibers	64.04 ± 2.83	18.60 ± 1.62	4.90 ± 0.69	[28]
Palm Oil Empty Fruit Bunch Fiber	36.7	35.8	18.6	[29]
*Ficus Macrocarpa* Bark	48.4	12.2	14.3	[30]
Sugarcane Bagasse	42.40	25.20	19.70	[31]

**Table 4:** Comparison of binding mechanisms and key properties of common pharmaceutical polymers in tablet formulation.

Polymer	Binding Mechanism	Key Controlling Properties	Advantages	Limitations	
HPC (Hydroxypropyl Cellulose)	Hydrogen bonding, plastic deformation, film formation	DS, viscosity, MW	Non-ionic, multifunctional, robust binding	Cost vs. native cellulose	[38]
HPMC (Hydroxypropyl Methylcellulose)	Hydrogen bonding, gel formation	Polymerization degree and viscosity	Controlled release capability	Higher gel strength may slow disintegration	[51]
PVP (Polyvinylpyrrolidone)	Solid-bridge formation	Molecular weight, solubility, viscosity	Strong binding at low levels	Synthetic origin, hygroscopic	[52]
MCC (Microcrystalline Cellulose)	Mechanical interlocking	Particle size, crystallinity	Excellent compressibility	Limited wet- binding	[53]

**Table 5:** Overview of hydroxypropyl cellulose (HPC) grades, processing methods, and corresponding drug release behaviors.

HPC Grade/Structural Parameter	Processing Method	Resulting Processing Property	Drug Release Behavior	Evidence Basis	References
Low viscosity (Klucel™ LF, EF, ELF; Mw ~95,000)	Direct compression/ wet granulation	High water solubility, rapid dispersion, maintains tablet hardness	Immediate release, fast disintegration	Commercial HPC	[58,59]
High viscosity (Klucel™ MF, HF)	Hot-melt extrusion (HME)	Gel-layer formation upon hydration	Sustained release over 8–12 h	Commercial HPC	[60,61]
Low-substituted HPC	Tablet coating	Taste masking moisture protection, improved mechanical strength	Compaction and disintegration	Commercial HPC	[50]
HPC matrix (general DS/MS)	Matrix tablet formulation	Hydration-driven gel layer, non fickian transport	Korsmayer–Peppas model, release exponent *n* = 0.5–0.8	Commercial HPC	[62]
HPC + chitosan	Interpenetrating polymer network (IPN)	Increase matrix rigidity	Controlled release of chlorthiazide and amoxicillin	Commercial HPC	[54,57]
Klucel™ LF vs. Klucel™ GF	HME-FMD 3D printing	Viscosity grade determines filament extrudability	LF: faster release (f_2_ > 70 vs. commercial benchmark); GF: sustained release (~8 h), comparable to marketed paroxetine tablets	Commercial HPC	[24,63]
SCB-derived HPC (DS 1.87)	Not yet evaluated in any tablet or matrix system	NR	NR	Direct SCB-DERIVED HPC data not available	[33]

**Table 6:** Comparative properties of hydroxypropyl cellulose (HPC) derived from sugarcane bagasse and wood pulp for pharmaceutical applications.

Parameter	Unit	HPC from Sugarcane Bagasse	HPC from Wood Pulp (Commercial Klucel™)	References
Source	-	Agro-industrial residue	Virgin wood pulp	[65,66]
α-Cellulose Content	%	42–50	90–95	[31]
Degree of Substitution (DS)	-	1.87	1.8–2.5	[33]
Crystallinity Index (CrI)	%	NR	80–87	[47]
Hydration Rate	-	NR	Moderate	[67]
Viscosity	mPa.s	NR	150–400	[39]
Particle Size	μm	NR	3.08	[22]
Tablet Hardness	kPa	4.0–4.5	4.5–5.0	[22]
Binding Force	MPa	NR	1.2–2.8	[45]
Estimate Finished-HPC Production Cost	(USD/kg)	(Not yet established, priority research need; see Section 3.15)	18–25	[49]
Sustainability	-	High (renewable byproduct)	Moderate (virgin wood)	[65]

NR = not yet reported in the primary literature for SCB-derived HPC; these parameters represent priority research needs identified in this review. Lower CrI → higher water accessibility → faster hydration/gel formation → influences disintegration or gel-controlled release.↑ DS → ↑ viscosity → ↑ polymer bridge formation → ↑ binding ability, whereas ↑ particle size → ↓ surface area → ↓ binding efficiency.

**Table 7:** The application of HPC in pharmaceutical research.

Application Category	Formulation	Drug	Function	Evaluation	Citation
a. Oral Drug Delivery and Tablet Applications	HPC modified to thiomer for mucosal DDS	Macromolecule drugs (e.g., proteins)	Mucoadhesive and permeation enhancement	Mucoadhesion test, DSC, swelling, thiol content	[68]
	HPC-based amorphous solid dispersion	Felodipine	Improve the solubility of a poorly soluble drug	Drug release, stability	[2]
	Natural polymer additives (HPC, HPMC, SLS) in clozapine crystallization	Clozapine	Modify the crystal form and morphology	UV–Vis, SEM, XRD, molecular simulation	[69]
	HPC grades (LF and EXF) for foam granulation	Not specified	Study of foam granulation for drug loading	Granule compactibility, size distribution	[70]
	HPC film and matrix in sustained release tablets	Hydrochlorothiazide	Binder and controlled-release modifier	Cloud point and disintegration affected by the substitution pattern	[71]
	Tablet with HPC from Betung Bamboo	Not specified	Binder	Good tablet properties: hardness 4.11 KPa, disintegration time 24.88 s	[22]
	Spray-dried HPC beads	Not specified	Controlled release excipient	Tunable release depending on moisture, size, and substitution level	[3]
	Hydroxypropyl cellulose in generic drugs	Generic drugs with hydroxypropyl cellulose	Fixed drug eruption caused by generic drugs	Cytotoxicity, patient case studies, and drug reaction tests	[72]
	Curcumin-loaded hydroxypropyl cellulose nanoparticles	Curcumin	Determine the pharmacokinetic parameters of curcumin	Pharmacokinetic analysis, drug bioavailability comparison	[73]
	Hydroxypropyl cellulose (HHPC, LHPC) oral administration	Not specified	Therapeutic potential for treating acute colitis	Pathological damage, inflammation mediators, and gut flora analysis	[74]
	Metformin hydrochloride coprocessed with HPC-L	Metformin hydrochloride	Improve the compressibility, stability, and flow properties of MET	Moisture sorption, compressibility, flowability, and dissolution	[59]
	Hydroxypropyl cellulose with HPMC for ITZ formulation	Itraconazole	Increase bioavailability of poorly soluble drug	Dissolution studies, bioavailability studies, DSC, XRPD	[75]
	Bioadhesive patch of HPMC E50 and Eudragit RS 100 for glibenclamide	Glibenclamide	Transdermal drug delivery for sustained release	Release profiles, permeation, zero-order kinetics	[76]
	L-HPC grades LH32, LH21, LH11, and LHB1	Not specified	Evaluate compaction and disintegration behavior in drug formulations	Compaction tests, rheological analysis, surface interaction studies	[77]
**Application** **Category**	**Formulation**	**Drug**	**Function**	**Evaluation**	**Citation**
b. 3D Printing and Controlled Release Systems	HPC-g-PAAm and chitosan microspheres	Amoxicillin trihydrate	Controlled drug release system	FTIR, SEM, XRD, DSC, release studies	[54]
	HPC with coformers in ternary dispersions	Model drugs	Improve drug stability in solid dispersions	COSMO-RS modeling, DSC, XRD, confocal microscopy	[78]
	Cannabidiol-loaded micelles in HPC gel	Cannabidiol	Controlled drug delivery	Rheological tests, drug release, cytotoxicity	[25]
	Rosemary essential oil in cellulose acrylate coacervates	Rosemary Essential Oil	Eco-friendly drug delivery system with essential oils	Thermodynamic analysis of coacervate formation	[79]
	Ternary solid dispersions of drugs with HPC and coformers	Various drugs (not specified)	Stabilize challenging drugs in ternary solid dispersions	In silico modeling, physical stability tests	[78]
	Hydroxypropyl cellulose with methyl cellulose and hydroxypropyl methylcellulose	Various drugs for controlled release	Control the sustained release of drugs via hot melt extrusion	Drug release under various conditions, rheological studies	[23]
	Amorphous solid dispersions using hot melt extrusion with HPC	Not specified	Investigate the effect of HME on moisture sorption properties	Moisture sorption isotherms, impact on stability and bioavailability	[80]
	Paroxetine in hydroxypropyl cellulose (HPC)-based formulations for 3D printing	Paroxetine	Create 3D printable formulations for controlled release of paroxetine	Release profiles, printability, and mechanical strength in 3D printing	[24]
	Hydroxypropyl cellulose (HPC) formulations for 3D printing with indomethacin or theophylline	Indomethacin, Theophylline	Optimize rheological properties of drug-polymer systems for 3D printing	Rheological behavior, printability, and thermal stability	[67]
	Methacrylated hydroxypropyl cellulose (MAHPC) for 3D printing and photocrosslinking	Not specified	Develop stable 3D printed parts using photocrosslinking of hydroxypropyl cellulose	Rheological properties, photocrosslinking kinetics, and stability of 3D parts	[81]
	Cholesteric HPC with in situ photo-crosslinking for 3D printing	Not specified	Enable tunable photonic structures for 3D printed objects with structural color	Rheological measurements, UV crosslinking kinetics, printability and microstructure	[21]
	Chitosan-HPC microspheres for chlorothiazide encapsulation	Chlorothiazide	Controlled release of chlorothiazide with biocompatibility	Encapsulation efficiency, swelling behavior, drug release	[57]
	HPC for drug encapsulation with temperature-responsive properties	Not specified	Temperature-responsive release for efficient encapsulation	Encapsulation efficiency, diffusion, release control	[82]
**Application** **Category**	**Formulation**	**Drug**	**Function**	**Evaluation**	**Citation**
c. Biomedical and Wound Healing Applications	Electrospun HPC composite for wound dressings	Not specified	Wound exudate absorption and antibacterial effect	SEM, tensile test, WVTR, antibacterial assay	[83]
	PU-HPC-βCD composite prepared via electrospinning	Not specified	Improved biocompatibility and physical properties	FTIR, SEM, DVS, biocompatibility tests	[84]
	Cinnamoyl-modified HPC	Not specified	Photocrosslinked material for biomedical applications	DSC, TGA, UV–Vis, cell test	[85]
	PVA/SA composite hydrogel with HPC fibers	Not specified	Ionic conductive hydrogel for nerve replacement	Tensile strength, ionic conductivity, and wear resistance	[86]
	Composite HPC/CMCS hydrogel	Imine-linked anticancer agents	Self-healing pH-responsive carrier	Chemical bond with drug; slow release in neutral, faster in acidic	[18]
	HPC/PVA composite hydrogel	Not specified	Improve mechanical strength and toughness in hydrogels	Mechanical strength, fatigue resistance, and conductivity	[87]
	HPC-based vitrification solution	Not specified	Cryopreservation of oocytes	Survival rates, fertilization rates, and embryo quality	[88]
	Chitosan/liquid crystal composite hydrogel	Not specified	Provide a biomimetic substrate for tissue engineering	Cell compatibility, fibroblast adhesion, and proliferation	[89]
	PCL/HPC/n-ZnO with Melilotus Officinalis	Not specified	Improve wound healing through nanofibrous structure and drug release	Fiber diameter, mechanical properties, wound closure index	[90]
	Oxidized hydroxypropyl cellulose with Jeffamines	Not specified	Design injectable, thermo-responsive hydrogels	Rheology, thermal response, and self-healing characteristics	[91]
	HPC hydrogel with OEO-loaded Pluronic micelles	Not specified	Topical treatment for melanoma with sustained release	Cytotoxicity, antiproliferative activity against melanoma cells	[92]
	Hydroxypropyl cellulose (HPC) and Pluronic F68 blends for tissue engineering scaffolds	Not specified	Develop biodegradable mucoadhesive hydrogels for tissue engineering	Biocompatibility, mucoadhesivity, biodegradability, and mechanical properties	[93]
	Acrylic acid and hydroxypropyl cellulose polymerized via free radical polymerization	Not specified	Synthesize and characterize hydrogels for biomedical applications	Polymerization efficiency, swelling tests, drug release profiles	[94]
	Hydroxypropyl cellulose ophthalmic insert (LACRISERT^®^)	LACRISERT^®^ (Hydroxypropyl Cellulose Ophthalmic Insert)	Improve patient-reported symptoms of dry eye syndrome with therapeutic insert	Patient-reported outcomes, clinical outcome assessments, eye care professional evaluations	[95]
	HPC membranes via salt-induced gelation	Not specified (membrane scaffold)	Tissue engineering scaffold, moisture barrier	BHN, gel thickness, erosion, SEM	[96]
**Application** **Category**	**Formulation**	**Drug**	**Function**	**Evaluation**	**Citation**
d. Film-Forming, Coatings and Packaging	Modified HPC through esterification to enhance hydrophobicity	Not specified	Formation of cellulose esters with improved hydrophobicity for coatings and films	Crystallinity test, FTIR, contact angle measurement	[19]
	HPC derivatives for reflective cholesteric films	Not specified	Used in eco-friendly optical reflective films	Bragg reflection, SEM	[97]
	HPMC-g-Poly(ethyl acrylate) via KPS grafting	Not specified	Tablet coating, humidity resistance	FTIR, SEM, TEM, DLS, TGA	[98]
	CNC modified with HPC	Not specified	Fabricate functional films	UV–Vis, CD spectra, TGA, SEM	[99]
	Hydroxypropyl cassava starch with agar and maltodextrin	Not specified	Create edible packaging with improved mechanical properties	Mechanical strength, water vapor permeability, and crystallinity	[100]
	Hydroxypropyl cellulose with chitosan nanoparticles	Not specified	Improve the properties of HPC and offer antifungal activity	Tensile strength, optical properties, and antifungal activity	[101]
	HPC photonic structures by nanoimprinting	Not specified	Fabricate photonic and plasmonic structures	Optical properties, material stability, and photonic applications	[102]
	Silver nanoparticles loaded onto HPC polymer	Not specified	Provide antimicrobial protection for linen textiles	Antimicrobial activity, mechanical and chemical property tests	[103]
	HPMC-Magnetite carbon dots composite film	Not specified	Food safety monitoring using color-changing composite films	FTIR, fluorescence, pH sensitivity, bacterial detection	[104]
	Oxidized HPC cross-linked with polyamines	Not specified	Enhance paper wet strength with low polymer amounts	Wet tensile strength, polymer distribution in paper	[105]
	Polypyrrole in HPC emulsion polymerization	Not specified	Polymerize polypyrrole in HPC for electrical conductivity	Particle morphology, electrical conductivity	[106]
	HPC-based GPE in DSSC with ionic liquid MPII	Not specified	Prepare gel polymer electrolytes for DSSC applications	Ionic conductivity, structural characterization, energy conversion efficiency	[107]
	Hydroxypropyl cellulose (HPC) with nanodiamonds for flexible electronics	Not specified	Develop sustainable nanocomposites for flexible electronics	Electrochemical performance, film stability, and printability	[108]
	Hydroxypropyl cellulose (HPC) with ethylene glycol for colorimetric temperature sensing	Not specified	Detect and visualize wide temperature ranges using colorimetric sensors	Temperature response, ranges from subzero to above room temperature	[109]
	Cellulose nanocrystals (CNCs) in hydroxypropyl cellulose (HPC) suspensions for films	Not specified	Create flexible, colored composite films with cellulose nanocrystals for various applications	Optical properties, film stability, and mechanical behavior	[110]
	Hydroxypropyl cellulose ester for organogels	Not specified	Fabricate functional organogels for bio-based applications	Swelling behavior, photo-crosslinking efficiency	[111]
	Cholesteric HPC films with temperature and humidity response	Not specified	Color-tunable films for responsive optical applications	Optical properties, pitch values, thermal and humidity testing	[112]
**Application** **Category**	**Formulation**	**Drug**	**Function**	**Evaluation**	**Citation**
e. Green Materials, Environmental and Recycled HPC	Synthesized HPC from waste paper	Not specified	Utilization of cellulose waste as a pharmaceutical material	FTIR, NMR, XRD, SEM, rheology	[34]
	Electrospun HPC fibers in ethanol/isopropanol	Not specified	Template for SnO_2_ nanofiber synthesis	SEM, fiber morphology	[113]
	Alpha cellulose from sugarcane bagasse, cationized	Not specified	Cationized cellulose for the paper industry	ANN modeling, FTIR, XRD, SEM	[65]
	HPC, nano-silica, hydrophobic nano-silica for ceramic consolidation	Not specified	Eco-friendly ceramic protection material	SEM, EDX, contact angle, mechanical tests	[114]
	HPC-capped silver nanoparticles	Not specified	HPC as capping agent for nanoparticles	UV–Vis, XRD, TEM	[115]
	Cellulose-based products, pulp	Not specified	Cellulose pulp production and export	Study of cellulose export and government support	[116]
	HPC/MoS2 composite for europium removal	Not specified	Remove europium from aqueous solutions	Adsorption capacity, surface interaction with Eu(III)	[117]
	HPC as a feed additive for all animal species with no minimum or maximum content	Not specified	Consider safety for animal consumption, efficacy as an emulsifier, stabilizer, and thickener	Toxicity, safety, and efficacy for animal feed additives, environmental impact	[118]
	HPC in aqueous foams for temperature-responsive applications	Not specified	Temperature-responsive foams for smart material applications	Thermo-responsive behavior, dynamic light scattering, rheological testing	[119]
	All-cellulose composite laminates from wood-based textiles with TEMPO-oxidized nanocellulose	Not specified	Create high-strength and thermal stable composite laminates from wood textiles	Tensile strength, elongation, thermal stability, and interface property measurements	[120]
	Oil palm EFB cellulose modified to hydroxypropyl cellulose	Not specified	Convert waste to useful biopolymer with enhanced solubility and application in food	MS, viscosity, crystallinity, purity of HPC from EFB	[121]
	HPC and nanocellulose for wood consolidation	Not specified	Consolidate and strengthen wood using HPC and nanocellulose composites	Wood sample consolidation tests, SEM, FTIR, and mechanical property analysis	[122]
	Hydroxypropyl cellulose synthesized via etherification of alpha-cellulose	Not specified (polymeric excipient)	Thickener, binder, stabilizer, emulsifier	DS, viscosity, IR, SEM, TGA/DTA, WAXD	[123]
	Hydroxypropyl cellulose synthesized from bamboo cellulose	Not specified (polymeric excipient)	Pseudoplastic thickener, stabilizer	Hydroxypropoxyl content, viscosity, SEM, IR	[22]
	Hydroxypropyl sago starch via low concentration propylene oxide	Not specified (starch-based excipient)	Thickener, food stabilizer	MS, swelling power, thermal analysis, SEM	[124]
	Hydroxypropyl cellulose from deinked waste paper	Not specified (recycled polymer)	Thickener, stabilizer, film-former	DS, NMR, IR, XRD, SEM, viscosity	[34]
	Magnetic HPC-g-PAA porous spheres	Not specified	Heavy metal ion removal from water	Adsorption capacity, kinetics, reusability	[125]
**Application** **Category**	**Formulation**	**Drug**	**Function**	**Evaluation**	**Citation**
f. Fundamental, Analytical, and Computational Studies	Not specifically formulated; focus on characterization	Oral DDS (not specified)	Enhanced thermal stability and crystallinity	XRD, DSC, TGA, CHNSO	[126]
	Cellulose esters and hydroxypropyl cellulose esters	Not specified	Investigate crystallization and phase transitions	DSC, X-ray scattering	[127]
	Cellulose-based hydrogels for flexible sensing	Not specified	Flexible, biocompatible sensing materials	Review of hydrogel types, applications	[43]
	Hydroxypropyl cellulose gel	Phenylalanine, Alanine	Chiral molecule detection	CD spectra, color change, gel stability	[20]
	Self-assembled cellulose derivatives	Not specified	Drug/gene delivery, biosensing, bioimaging	Review of methods, applications	[128]
	HPC and PVDF blend	Not specified	Study phase separation in polymer blends	Phase diagrams, TEM, XRD, TGA	[129]
	HPC-lignin blends	Not specified	Study of miscibility in polymer blends	Morphology, melting point depression	[130]
	Nanocellulose and composites	Not specified	Medical applications like tissue engineering	Literature review on extraction, dissolution	[131]
	HPC esterified by decanoyl chloride	Not specified	Enhance liquid crystal behavior and thermal stability	FTIR, NMR, TGA, polarizing microscope, thermal stability tests	[132]
	Lignin-modified HPC blends	Not specified	Blend morphology and properties of HPC-lignin mixtures	Ultrasonic method, phase transition analysis	[130]
	Lyotropic liquid crystalline HPC solution	Not specified	Study phase transitions of HPC in solution	Phase transition analysis using ultrasonic variables	[133]
	Liquid crystalline polymers of HPC with n-hexanoyloxypropyl group	Not specified	Study the phase transition behaviors of HPC in bulk	Ultrasonic method to detect phase transition	[133]
	TEMPO-oxidized HPC solution	Not specified	Investigate cholesteric liquid crystal properties of HPC	X-ray diffraction, NMR, impact of substituents on liquid crystal structure	[134]
	HPMC with controllable molecular mass and viscosity	Not specified	Control the rheological properties of HPMC solutions	Viscosity, gelation temperature, rheological behavior	[135]
	Various molecular weights of HPC in oil emulsions	Not specified	Emulsion stabilization and enhancement of viscosity	Emulsion drop size, molecular weight impact, viscosity	[51]
	Oxidized HPC with TEMPO and sodium periodate	Not specified	Introduce functional groups in HPC for further modification	Oxidation degree, chemical structure, crystallinity, and thermal stability	[47]
	HPC in ionic liquid for mesophase and optical control	Not specified	Control the cholesteric mesophase structure in HPC	Mesophase structure, optical properties, and electrical control	[136]
	HPC in cholesteric liquid-crystalline phase	Not specified	Study dynamic rheology in nematic and cholesteric phases	Rheological responses, orientation relaxation time	[137]
	HPC in aqueous solution with salt additives	Not specified	Control phase-separation behavior and optical properties in HPC solutions	Spectrophotometry, NMR, ionic effect on mesophase	[136]
	HPC in pyridine, ethanol and other organic solvents	Not specified	Investigate cholesteric mesophase formation in solvents	Microscopic observation, cholesteric pitch measurement	[138]
	Cellulose-based materials with temperature, light, electrical, and magnetic responses	Not specified	Develop smart materials with various stimuli-responses for technological applications	Performance in soft robots, actuators, and artificial muscles	[139]
	Modified cellulose derivatives with functional groups for improved solubility and performance	Not specified	Enhance cellulose properties for industrial applications and sustainability	Solubility, environmental impact, and physical properties of modified cellulose	[140]
	Hydroxypropyl cellulose (HPC) with various salts and ions in aqueous solutions	Not specified	Study the isomerism of hydroxypropyl cellulose in solution for a better understanding of its properties	IR spectroscopy, phase transitions, and solubility studies	[141]
	Chiral HPC and CNC in liquid crystal phase for photonic elastomers	Not specified	Enhance material properties for use in photonic and manufacturing applications	Synchrotron X-ray, neutron small-angle scattering, photonic characterization	[142]
	HPC as a tuning additive in DL-methionine crystallization	DL-Methionine	Regulate crystallization behavior of DL-methionine, improving industrial processes	Crystallization behavior, thermodynamic calculations, kinetic analysis, and crystal morphology	[143]
	HPC in aqueous solutions, forming a cholesteric phase	Not specified	Study the liquid crystal behaviors and optical properties of HPC in solution	X-ray diffraction, humidity-driven instability, films and fibers behavior analysis	[144]
	HPC with variability in the substitution pattern affecting its properties	Not specified	Study the effect of substitution pattern on HPC properties for applications	Rheological, surface, and thermal properties, comparison between different HPC formulations	[3]
	HPC in photonic and plasmonic crystal formations	Not specified	Create photonic crystals and flexible plasmonic structures	Spectral response to light, fabrication of devices	[145]
	Modified hydroxypropyl cellulose via grafting for various applications	Not specified	Enhance functional properties for various industrial applications	Physical properties, grafting efficiency, solubility tests	[146]
	1–8% hydroxypropyl cellulose solution with surfactant SDS	Not specified	Study of rheological properties and gelation induced by surfactant	Viscosity analysis, gelation tests, and critical micelle concentration evaluation	[147]
	Hydroxypropyl cellulose and propylene glycol blend for LCP system	Not specified	Examine adhesive properties and gelation characteristics	Rheological measurements, adhesive testing at different contact times	[148]
	HPC in aqueous solution for hydroxypropoxy group assay	Not specified	Quantify hydroxypropoxy groups in hydroxypropyl cellulose using USP method	USP titration for hydroxypropoxy group content, purity analysis	[149]

**Table 8:** Regulatory considerations and consequences for bagasse-derived hydroxypropyl cellulose (HPC) in pharmaceutical applications.

Aspect	Current Regulatory Expectation	Implications for SCB-Derived HPC	Key References
Pharmacopeial status	HPC monographs exist in USP–NF, Ph. Eur., JP	Must comply fully with existing HPC specifications	[153,154,155]
Cellulose source	Implicitly wood-/cotton-based	Alternative source requires equivalence demonstration	[156]
DS and viscosity	Defined acceptance ranges	Must fall within compendial limits	[153,157]
Substitution pattern (substituent distribution)	Not always explicitly specified in compendial monographs but affects functional equivalence	Requires NMR-based characterization to confirm structural equivalence beyond average DS	[41,154]
Impurity profile	Limits for heavy metals, residue on ignition, solvents	Enhanced control for agro-residue-derived impurities	[158,159,160]
Batch-to-batch consistency	Required for excipient qualification	Greater emphasis on feedstock standardization	[161,162],
GMP requirements	Pharmaceutical-grade manufacturing	Full GMP compliance mandatory	[161]
Regulatory pathway	Excipient equivalence or prior-use justification	Comparative data vs. commercial HPC required	[163,164]
Pesticide residues	Limits per pharmacopeial general chapters	Required due to agricultural origin of feedstock; not applicable to wood/cotton-pulp-derived HPC	[165,166]
Microbial limits	Total aerobic microbial count, absence of specified pathogens	Greater scrutiny warranted given agro-residue origin and potential field/storage contamination	[167]

## Data Availability

No new data were created or analyzed in this study.
